# “I was raped by the broker on the first day of my arrival in the town.” Exploring reasons for risky sexual behavior among sexually-active unmarried young female internal migrants in Ethiopia: A qualitative study

**DOI:** 10.1371/journal.pone.0242176

**Published:** 2020-11-13

**Authors:** Ararso Baru, Ikeola A. Adeoye, Adeyemi O. Adekunle

**Affiliations:** 1 Institute of Life and Earth Science (Including Health and Agriculture), Pan African University, Ibadan, Nigeria; 2 College of Medicine and Health Sciences, Arbaminch University, Arbaminch, Ethiopia; 3 Research and Collaboration Department, Slum and Rural Health Initiative (SRHIN), Country Director to Ethiopia, Addis Ababa, Ethiopia; 4 Department of Epidemiology and Medical Statistics, Faculty of Public Health, College of Medicine, University of Ibadan, Ibadan, Nigeria; 5 Department of Obstetrics and Gynecology, Faculty of Clinical Medicine, College of Medicine, University of Ibadan, Ibadan, Nigeria; Karolinska Institutet, SWEDEN

## Abstract

**Background:**

Studies revealed that internal migrants are one of the most vulnerable groups for poor sexual and reproductive health (SRH) information and services. Risky sexual behavior (RSB) is a threat to public health and might lead to serious health problems such as unintended pregnancy, abortion, and sexually transmitted infections (STIs) including HIV/AIDS. The reported prevalence of RSB among young female internal migrants in Ethiopia was as high as 70.3%. This requires in-depth understanding of the underlying cause. So, this study aimed at exploring reasons for RSB among sexually-active unmarried young female migrants in Ethiopia.

**Methods:**

A descriptive qualitative study was conducted using focus group discussions among sexually-active young female migrants working Burayu town. The focus group discussions were done in the local languages of participants (Afaan Oromo and Amharic). The recorded data were transcribed verbatim and translated into English for analysis and presentation in the study. The data were coded and Atlas.ti 7.5 software packages were used for data analyses. Then, the findings were thematically organized and analyzed using content analysis.

**Results:**

This study revealed that poor socio-economic status, social media indulgence, rape, substance use, poor knowledge of condom use, unfavorable attitude toward condom use, misconceptions about emergency pills, and the nature of the new environment and work place were responsible for RSB among internal migrants. The participants described that the migrants’ economic conditions and workplace sexual violence are pushing them toward engaging in unprotected sex, being sexually abused, commercial sex, and transactional sex.

**Conclusions:**

Internal migrants’ sexual behavior is a complex process influenced by multiple interrelating systems. We have explored a set of factors namely poverty, pressure and sexual abuse from brokers, sexual exploitation and abuses against domestic workers by their bosses, indulgence in social media, sexting, inadequate knowledge, and unfavorable attitude toward condom use that led young female internal migrants to risky sexual practices. An intervention to promote safe sex targeted to this population is urgently needed with a focus on an intervention to eliminate misconceptions about condoms, increase proper condom use, and end sexual violence. Moreover, a relevant policy is needed to safeguard internal migrants from sexual exploitation and abuses at their work place.

## Background

Ethiopia is one of the Sub-Saharan Africa (SSA) countries with an estimated 80 percent of the population inhabiting rural areas [1]. Recently, the urbanization rate in Ethiopia has been rapidly increasing at about 5.4 percent per year and by 2028 the urban population will reach 30 percent [2]. Over the last few decades, rural to urban migration in Ethiopia rose due to economic, climatic and political factors which include drought, war, political turmoil, forced migrations, and poverty [3, 4]. Internal migration has a lion’s share of the rapid growth in urbanization [5]. However, it also plays a crucial role in shifting the HIV/STI epidemic by broadening social and sexual mixing [6–9].

Studies revealed that internal migrants are one of the most vulnerable groups for poor sexual and reproductive health (SRH) information and services [10, 11]. Risky sexual behavior (RSB) is a threat to public health and might lead to serious health problems such as unintended pregnancy, abortion, and sexually transmitted infections (STIs) including HIV [11]. Even though RSB may lead to unfavorable outcomes, the reported prevalence of RSB among young female internal migrants in Ethiopia was as high as 70.3% [12].

Different factors that make young female internal migrants vulnerable to RSB have been reported. Firstly, nearly two-thirds of the internal migrants in Ethiopia are young and economically disadvantaged groups with little education and limited skills [13, 14]. Uneducated internal migrant women may end up with few livelihood options in urban areas particularly when competing with a better-educated urban labor force [15, 16]. Secondly, economic pressures and limited employment opportunities in urban areas are more likely to push internal migrant women to RSB [17, 18]. For example, a study in Ethiopia revealed that young female migrants spent more time in unpaid domestic work and they work in physically and sexually abusive environments, which may expose them to high-risk sexual practices [19]. Thirdly, internal migrants have limited knowledge and access to reproductive health services particularly during the early years of their stay in urban areas due to the new environment and restricted social contacts [11, 20, 21].

Most studies conducted on RSB in Ethiopia were mainly focused on key population such as female sex workers, secondary school students, university students, adolescents, youth, and substance users [12, 14, 22–25]. Young female internal migrants are a particular group whose sexual behavior has been given less attention in Ethiopia. Therefore, this study aimed at exploring reasons for RSB among sexually-active unmarried young female internal migrants in Burayu town, Ethiopia using a qualitative study technique.

## Methods and materials

### Study design and setting

A descriptive qualitative study was carried out between April and June 2019 in Burayu town. The town is located about 15 kilometers toward the Western fringe from the center of Ethiopia’s capital, Addis Ababa. The town has six kebeles (the smallest administrative unit in Ethiopia) which includes Gafarsa Nonno, Gafarsa Guje, Gafarsa Burayu, Malka Gafarsa, Burayu Kata, and Lakku Kata. Burayu town is one of the industrial zones in Ethiopia and has rapidly growing infrastructures, electricity, telecommunication, and banking services. However, the town is characterized by inadequate transportation, health care services, and youth recreational facilities [26]. The town's proximity to Addis Ababa attracts a substantial number of internal migrants, as a result, there are many young people vulnerable to unemployment and socio-economic crisis [26, 27].

### Study population

The study was done among participants recruited from factories, restaurants, coffee vendors, hotels, cafeterias, and bars in Burayu town based on their age, sex (only female), sexual history, and migration status. Eligibility criteria included being internal migrants, female, aged between 15–24 years, unmarried, experienced at least one account of penetrative sexual intercourse over the last six months, and consented to take part in the study. The exclusion criteria consisted of unwillingness to take part in the study, as well as the inability to respond due to medical and surgical illness.

### Data collection method and procedures

Focus group discussion (FGD) helps to ethically gather richer data and culturally relevant information [28, 29]. One of the advantages of FGD compared to individual interview is that it can generate more ideas and yield deeper insights into the problem under investigation by encouraging participants to talk and interact with each other [30]. In addition, FGD helps to collect indirect evidence on sexual behavior. For example, people can report others behavior or they can talk about themselves under the guise of discussing others behavior during a FGD [29].

Literature suggested that the size of the FGD group should be between six and twelve participants so that the group is small enough for all members to talk and share their thoughts, and yet large enough to create a diverse group [31, 32]. Nowadays, qualitative research experts believe that the concepts of data saturation are a subjective phenomenon and they suggest the concept of information power to explore adequate information on qualitative research than theoretical saturation concepts [33]. In accordance with these recommendations, this study adequately achieved information power that generated new knowledge and met the aims of the study using four focus group discussions (FGD) with eight participants in each group. Participants were initially recruited using active and passive recruitment strategies. Active recruitment was done through randomly asking potential candidates from community-based entertainment and non-entertainment venues such as coffee drinking shops, restaurants, bars, and factories while passive recruitment was done through distributing fliers to these venues. Based on eligibility criteria the eligible participants were screened for the study. In addition, the eligible participants were asked to refer another participant (Snow-ball sampling method) to the study. Snow ball sampling method helps researchers to gain access to the target population [34]. The choice of a new subject was guided by the aim and objectives of the study. Moreover, participants were compensated 200 ETB (about 6.5 USD) and provided refreshments during the meeting.

With regard to the place of participant recruitment, six main occupational clusters (factories, restaurants, coffee vendors, hotels, cafeterias and bars) that employ young female migrants were identified. Five participants were purposefully recruited from each cluster except for factories and restaurants (six of each).

An English focus group discussion guide was adopted from the tool developed by WHO to assess young people’s sexual behavior with modification to internal migrants and study setting [35]. The translation of the language followed the WHO’s recommendation on instrument translation [36]. Initially, the forward translation of the interview guide was done by the principal investigator, who is a health care professional and fluent in the languages of the participants. Then, expert and back translation were done to meet the aims of the study. Moreover, the guide was reviewed for cultural relevance by a local ethical review committee.

The FGDs were conducted in a favorable environment (a room that was free from noise] at community-based venues that were preferred by the participants. The discussions were held in either Amharic and Afan Oromo based on the participant’s language preference. The discussions were facilitated by the principal investigator (the first author) and two experienced research assistants. All facilitators speak the languages of the respondents and they were familiar with the local culture. The principal investigator (the first author) and two experienced research assistants facilitated the discussions. All facilitators speak the languages of the respondents, and they were familiar with the local culture. The principal investigator led the discussion while research assistants recorded field notes, operated the digital recorder, and observed group dynamics. Each FGD began with the facilitator discussing the purpose of the study. Ground rules such as respecting the views of each other, allowing only one person to speak at a time, and not to name people or organizations that harmed them were set prior to beginning the discussion. Additionally, study participants were encouraged to freely answer the questions, share their opinions and experiences during the discussions. Each FGD lasted 64 to 80 minutes.

### Data quality control

To establish the validity of the work, this study used the following techniques. The study adopted the FGD guide recommended to assess sexual behavior of young people by the WHO [35]. Similarly, a single FGD was done for pretesting to validate the FGD guide in Sabata town, which is also one of the major destinations of internal migrants in Ethiopia [27]. Pretested participants were similar to the study participants in terms of migration status, cultural context, and residential proximity to the study area. After pre-testing, some culturally sensitive words referring to sexual practices were omitted while some missed information was added to the FGD guide. For example, the types of sexual activities such as anal sex and oral sex were excluded after pretesting because participants were refused to disclose type of sex they had. In addition, questions about homosexual behavior were also excluded, because validation-step participants did not report this practice. This lack of disclosure may be because the behaviors were not practiced, or because pre-test subjects did not wish to disclose them. Moreover, participants with similar age group and employment structure were included in the same group to enhance the freedom of their thought expression.

### Data analysis

The recording of the FGDs was transcribed verbatim and translated into English for analysis and presentation in the study. Field notes from each focus group were independently reviewed by the facilitator and annotated with the transcripts. The data was coded and Atlas.ti 7.5 software package was used for data analyses. Then, the findings were thematically organized and analyzed using content analysis.

### Transferability of the finding

The potential transferability of the evidence generated by this study to other settings should be considered in view of the methods, settings, and context described in this study.

### Ethical consideration

Ethical clearance was obtained from the University of Ibadan /University College Hospital (UI/UCH) ethical review board (Certificate of Ethical Clearance No: 18/0550). Similarly, clearance was obtained from the Oromia Regional Health Bureau (ORHB) through PAULESI.

The purpose of the study was explained to the participants by the language they understand and verbal consent was obtained from each study participant before proceeding to the data collection. The respondents were informed that they had the right to be involved or refuse to participate in the study. In addition, the respondent had the right to withdraw from the study at any time during the FDG without needing to disclose the reason for dropping out. The participants were assured that the data would be handled exclusively by the investigators and they would remain completely anonymous in the report. They were also informed that they could read the transcripts and citations if they wanted. Furthermore, the confidentiality of the information obtained from each participant was maintained and the softcopy of the database was kept in a password protected computer by the principal investigator.

## Results

A total of four FGD were conducted with each group comprising eight participants. The total numbers of participants involved in the FGD were thirty-two. The participants mentioned various reasons for early sexual initiation and unprotected sexual intercourse. After thorough content analysis and review of the literature, the mentioned reasons were categorized and presented in four broad themes. These themes include socio-economic, personal, nature of the new environment, and social media and technology related factors. All these reasons given by FGD participants are interrelated.

### Theme 1: Socio-economic factors

#### Poverty is pressuring young female migrants to early sexual debut and transactional sex

Most of the FDG participants reported that they first had sex before the age of 18 years old and some participants even started as early as 13 years old. The participants felt that their economic condition pressured them to have their first sexual encounter at an early age. Some participants also initiate the first sexual intercourse after receiving gifts from their loved ones. One of the FGD participants remarked that:

*“…The day the only underwear pant I had torn and I asked my uncle’s wife to buy me a new one*. *However*, *she refused and I decided to contact the man who has been asking me out for a while (crying)*… *I remember it was on a Friday that I begged a shopkeeper in my area to allow me to make a phone call to the man using his phone*. *I then fixed an appointment with him and we agreed to meet on Sunday morning*. *On the first day we discussed our relationship and at the end of the date*, *the man gave me 200 ETB (which was about 7 USD)*. *I was very much excited and bought some important stuff such as sanitary pads and underwear with that money*. *On the second day*, *the man gave me some more gifts and this surprised me a lot*. *Following the gift*, *I did my first sexual intercourse and I was only 16 years old at that time”*. (Age 19, FGD3 participant).

#### Giving sex to support their families economically

The participants who came from economically disadvantaged families have the responsibility to provide financial support to their families who live back home. When their parents asked them for money, they sometimes borrow from friends or neighbors. In case they are unable to return the money on the appointed date, some of the men ask for sex. One of the FGD participants remarked that:

*“I have been helping my family (financially) who lives back home*. *Sometimes*, *I borrow money from the owner of the restaurant where I work*. *Whenever he wants sex he asks about the money he lent to me before*. *I can’t refuse sex because I don’t have money to pay him*. *So*, *I do anything he likes including sex without condoms to extend the time to return his money”*. (Age 20, FGD4 participant).

### Theme 2: Personal factors

#### Substances uses

Young female internal migrants felt that being away from their parents offered them an opportunity to explore different types of substance. They used these substances with the idea that they wouldn’t have to deal with the consequences of deviating from the norms of the society where they grew up and disappointing their parents. Some of them give sex to casual partners to get free khat and alcohol, which they believed facilitates the eventuality of high-risk sexual practice. It is believed that alcohol intake helps to soothe one’s high from the khat. As explained by an FGD participant:

*“I became addicted to khat (Catha edulis plant) at my current destination. After chewing khat, ‘cabsi’ (a local term used among khat chewers to express alcohol intake) is mandatory to fall asleep. Since I am a woman, I don’t worry to get sponsors for khat and beer. I simply give sex, even to a casual partner, and enjoy my life.” (Age 21, FGD 2 participant)*.

#### Limited knowledge on proper use of condoms

All FGD participants had heard about and seen condoms before. However, many of the participants had limited knowledge about how to correctly use condoms. A young teenager remarked that:

*“I occasionally use condoms with my boyfriend but I don’t know how to properly use it*. *I have never gotten any form of education on the proper way to use condoms*. *In my community*, *talking about sexuality is taboo*. *So*, *I don’t ask about that thing even to this day when I am not living in that community anymore* (where I grew up).*”* (Age 17, FGD 1 participant).

#### Perceiving condom use as a sign of distrusting their partner

Many participants in the study had a bad attitude toward condom use. The majority of the respondents said that they do not ask their partners to use condoms because such a question can irritate them (the men) and some men consider it an indicator of fake love.

*“I don’t use a condom with someone who I’m in love with*. *This is because I believe that it is a sign of distrust*. *My boyfriend also doesn’t like it*.*”* (Age 18, FGD 2 participant).

#### Men dominance on condom use decisions

Men are dominating their partner on condom use decision. The following quotations are illustrative of some participants’ discussions of relying on men to use a condom.

*“I prefer to use condom but my partner doesn’t like it*. *To please him*, *I have sex with him without using condoms*.*”* (Age 20, FGD4 participant).

*“My boyfriend doesn’t like to wear condom so he is the one in a position to decide whether to use it or not*. *On the first round of our sexual contact*, *we used condom*. *However*, *starting from the second round of the first day he refused to use condom and we are not using it anymore*.*” (*Age 23, FGD 4 participant).

#### Feeling of embarrassment to buy condom

Some people blamed their shyness for their reluctance to buy a condom for unprotected sexual intercourse. They explained that their shyness was related to cultural background. For example, simply saying the word “condom” which is taboo at the place they grew up affects their sexual behavior at the new destination. As one of them stated

*“I feel embarrassed to buy condoms*. *Forget about buying*, *I even fear the name condoms (laughing)*. *I remember the day we went out to buy condom with my boyfriend*. *We reached the pharmacy and we were scared to ask for condoms*, *then we asked for paracetamol instead of condoms*. *That day we had unprotected sexual intercourse*.*”* (Age 19, FGD 3 participant).

#### Poor knowledge and misconception about emergency pills

Some respondents also reported that they use emergency contraceptive pills regularly. There was a respondent who was misinformed by friends that emergency contraceptive pills can prevent both unwanted pregnancy and HIV transmission. As revealed by a young FGD participant:

*“I have been using condoms with my regular partner but later we shifted to a tablet that taken twice after coitus (Post pills)*. *I will tell you frankly that we* (her boyfriend and the woman) *were thinking that post pills prevent both HIV and pregnancy*. *I just learned about it today from this focus group discussion*.*”* (Age 20, FGD 1 participant).

### Theme 3: Nature of the new environment and workplace

#### The new environment and vulnerability to rape

The FGD participants reported that some young female migrants were raped after migration either by someone they knew before the incident or by a stranger—usually by brokers; this suggests the presence of violence against young female internal migrants. As stated by an FGD participant:

*“…My close friend and her brother welcomed me at a bus station*. *Since we grew up in the same village*, *I trusted them*. *I followed them to their house*. *She gave me water for bathing and we ate dinner together*. *I have been on the road for more than 12 hours*. *As a result*, *I felt tired and asked them that I wanted to sleep*. *The man left the room and I remained with his sister*. *Approximately*, *it was about 1*:*00 am that someone touched my body and I asked the identity of the person*. *I never expected the incident and it was my friend’s brother*. *He told me that he had been in love with me for a long time*. *What he said shocked me and I was unable to believe what was happening*. *I tried to search for his sister but she had already left the room*. *Since it was raining*, *no one heard my voice and he raped me that night*.*”* (Age 20, FGD1 participant).

In some instances, young women migrate to escape an arranged marriage. However, their new destination facilitates sexual abuse. One woman lamented her experience as follows:

*“I left home to avoid an arranged marriage and reached the bus station around 8*:*00 pm but I did not know anyone in the town*. *As soon as I exited from the bus station*, *someone asked me if I was searching for a bed*. *Then*, *I replied yes and agreed to follow him (the broker)*. *While heading to the place*, *I told the man that I am new to the place and wanted a job*. *Then*, *he (the broker) shook his head (shaking of the head is considered as showing sympathy for someone) and told me as he could get a job for me*. *Approximately*, *about 15 to 20 minutes later*, *we reached the place and he paid for the bed*. *Then*, *he went out to buy some food for me and we ate together*. *At midnight*, *the broker asked me to have sex with him and I refused because I had never done that thing (sex) before*. *However*, *I was not strong enough to protect myself and he raped me*. *It was that night I was forced to start the first sexual intercourse*.*”* (Age 18, FGD 4 participant).

#### Sexual violence in the workplace

Some women working in hotels, bars, or restaurants reported that the owners of the business ventures sometimes forced them to sleep with their customers and vandals for the sake of their business and safety. A young adult lamented as follows:

*“I have been working as a waitress in a small bar located in Burayu town*. *One day*, *after drinking a lot of beer from the bar*, *a locally known ‘duriye’ (the Amharic equivalent for vandal) asked me to sleep with him*. *But I refused and told him that I am not a commercial sex worker*. *I don’t know what the man (*the vandal) *communicated with the owner of the bar but they both came together and the bar owner commanded me to sleep with the man*. *He* (the bar owner) *said*, *‘the man can destroy what I* (the bar owner) *have here*, *so go and sleep with him otherwise I will fire you this night’*. *I don’t have anyone from the place so I preferred to be silent*. *The man took me to a bedroom and did everything he liked on me that night*.*”* (Age 21, FGD3 Participant).

#### Domestic worker sexual exploitation and abuse as a precursor for commercial sex engagement

Some young female migrants who were former domestic workers commenced commercial sex work due to sexual exploitation and abuse by their bosses. A former domestic worker remarked that:

*“…I started working as a housemaid in Burayu town but was repeatedly raped by the owner of the house. Whenever the woman (wife of the rapist) was not around*, *he would return home early to rape me. Sometimes, he would owe some money to silence me. After a while, I left and rented houses to get freedom from sexual and psychological abuse. Currently, I am working at the hotel as a janitor but the cost of living is tough and sometimes I do sex work to get supplementary income.”*

### Theme 4: Misuse of social media and modern communication technologies

#### Exposure to social media and its role in risky sexual behavior

Social media is easing communication. However, its misuse emerged as an important factor that impacts young female internal migrants’ sexual behavior. This is based on the reports given by most of the FGD participants. As stated by one of the FGD participants:

*“After coming to Burayu town*, *I got access to the internet and social networking such as Facebook and telegram which was not common in my village*. *It makes communication very easy*. *I always chat with several men per day over social media*. *I change my profile photo periodically and I believe it helped men to be attracted to me*.*”* (Age 18, FGD 4 participant).

The findings highlight the point that misuse of social media can lead young female internal migrants to sext, which may further lead them to unprotected sexual intercourse. The narrative of one of the participants revealed that *“Without social media*, *it could have taken even years for me to date someone*. *But I dated within a month of my arrival to Burayu town*. *I’m usually scared to talk to a person that I don’t know before physically but not on social media*. *Currently*, *I do not feel any embarrassment to share naked images through a private chat over social media with my partner*. *Sometimes*, *we do unprotected sex following sexting”* (Age 19, FGD 1 participant).

#### Social media and recent technologies as a tool for blackmailing and sexual abuses

Some FGD participants also reported that social media and recent technologies are exposing them to blackmailing and sexual abuses:

“*A friend of mine had a boyfriend who was from the same town as her but had migrated a long time ago*. *He captured video of her naked body and all sexual activities they had together without her knowledge*. *Later*, *he sent the video over Facebook to her*. *Sometimes*, *he threatens to send the video to her family as well*. *So*, *she had no choice other than giving sex and money to him when he needs them*. *However*, *she was unable to cope with sexual abuse and blackmailing*. *Finally*, *she gave money to his close friend and he stole her partner’s mobile phone*. *Then*, *she deleted the video from the phone and solved the issue (loudly laughing)*.” (Age 17, FGD 3 participant).

## Discussion

The main goal of this study was to get a deep understanding of the reasons for engaging in risky sexual behavior among sexually-active unmarried young female internal migrants in Ethiopia. Through focus groups, we identified four themes that provided a better understanding of reasons for risky sexual practices among young female internal migrants. These themes are (a)socio-economic factors, (b) personal factors, (c) nature of the new environment, and (d) social media and technology related factors.

Our finding revealed that almost all of the discussants in each FGD blamed economic conditions for an early sexual debut. Similarly, from earlier studies, being from an economically disadvantaged community more likely pushes young people to risky behavior such as transactional sex at a younger age [12, 37].

The FGD participants blamed alcohol and drug use for RSB in this study. A number of previous studies examined and revealed that substance use including alcohol could increase the likelihood of RSBs [38, 39]. The reason might be due to the nature of alcohol in decreasing attention to safe sex practices, altering rational decision-making, and increasing risk-taking behaviors [40, 41]. Similarly, the study participants blamed khat use as the reason for unprotected sexual intercourse among young female internal migrants. The relationship between khat use and RSB was also reported by previous studies in Ethiopia [25, 42].

A previous study conducted in Ethiopia reported that brokers forced the internal migrants for sex by taking advantage of the fact that the girls had no place to stay [43]. This study also found that some young female migrants were raped after migration either by someone they knew before the incident or by a stranger, but usually by brokers, and they were forced to have unprotected sexual intercourse.

The FGD suggests that domestic work is making young female internal migrants vulnerable to sexual violence. Some of the victims of sexual violence within domestic workplaces claimed that they started commercial sexual work as a means of escaping this sexual abuse and to support their daily living expenses. This was also previously reported by a study conducted in six regions of Ethiopia which found that 49 percent of former domestic workers began commercial sex work due to sexual abuse [44]. Further research should thoroughly explore the extent of sexual abuse among domestic workers and the association with commercial sex work.

This study found that a lot of taboo and particular beliefs surrounded the idea of condom use. For example, some women are scared to suggest using a condom because such a proposition might irritate their partner or be considered as an indicator of ‘fake love’ by some men. Additionally, young females may agree not to use condoms based on the belief that their partners might not enjoy it, as previously reported by other studies [45, 46].

Using internet, mobile phone technologies, and social networking sites emerged as important factors that influence young female internal migrant sexual behavior according to this study found. Technologies helped to overcome the communication barrier due to cultural factors. However, it is facilitating a tendency to have multiple sexual partners and other risky sexual practices [47, 48]. In addition, this study showed that sexting over social media is exposing young female internal migrants to unprotected sex, sexual abuse, and blackmailing. Similarly, sexting of any kind is associated with various forms of RSB according to a previous study [49].

Despite the fact that this study explored a range of major reproductive health issues related to sexually-active young female internal migrants in Ethiopia, it has several limitations. First, this study is based on the self-report of participants which are subject to recall bias. To minimize bias, questions were asked more than once, answers were repeated back to participants, and findings were reviewed at the conclusion of the FGDs. In addition, sexual experiences of young female internal migrants as recorded in our data may cause social desirability bias. We minimized this problem by implementing various interventions such as encouraging study participants to speak and repeatedly informing them of the purpose of the study. Although the study participants migrated from various regions of Ethiopia to Burayu town, the information used in this study may not be representative of unmarried rural-to-urban female migrants in other regions. Therefore, the finding of this study is limited to the context of the study setting. This calls for caution when generalizing the findings reported here to the context of other internal migrant’s destinations in Ethiopia.

## Conclusion

Young female internal migrants appear to be a core group involved in high-risk sexual behaviors in this study. Internal migrants’ sexual behavior is a complex process influenced by multiple interrelating systems. We have explored a set of factors namely poverty and economic insecurity, pressure and sexual abuse from brokers, sexual exploitation and abuses against domestic workers by their bosses, indulgence in social media, sexting, inadequate knowledge, and unfavorable attitude toward condom use that led young female internal migrants to risky sexual practices.

### Implications

The findings of this study can be used as a basis for further studies which may help to prevent or reduce RSB among young female internal migrants. An intervention to promote safe sex targeted to this population is urgently needed with a focus on an intervention to eliminate misconceptions about condoms and increase proper condom use. Strengthening government-accredited brokers should be thought of by policymakers to reduce the magnitude of sexual violence among this group. Moreover, a relevant policy is needed to safeguard internal migrants from sexual abuses and exploitations at their workplaces.

## Abbreviations

AB: Ararso Baru
AIDS: Acquired Immuno-deficiency Syndrome
AOA: Adeyemi O. Adekunle
FGD: Focus Group Discussion
HIV: Human Immuno-deficiency Virus
IAA: Ikeola A. Adeoye
RSB: Risky Sexual Behaviour
SRH: Sexual and Reproductive Health
STIs: Sexually Transmitted Infections
USAID: United States Agency for International Development
UNFPA: United Nations Fund for Population Activities
